# Avoiding the Next Silent Spring: Our Chemical Past, Present, and Future

**DOI:** 10.1021/acs.est.3c01735

**Published:** 2023-04-13

**Authors:** Hans Peter H. Arp, Dagny Aurich, Emma L. Schymanski, Kerry Sims, Sarah E. Hale

**Affiliations:** † Norwegian Geotechnical Institute (NGI), P.O. Box 3930, Ullevål Stadion, 0806 Oslo, Norway; ‡ Department of Chemistry, Norwegian University of Science and Technology (NTNU), 7491 Trondheim, Norway; § Luxembourg Centre for Systems Biomedicine (LCSB), University of Luxembourg, 6 avenue du Swing, 4367 Belvaux, Luxembourg; ∥ Environment Agency, Horizon House, Deanery Road, Bristol BS1 5AH, U.K.

**Keywords:** precautionary principle, persistent, chemicals, patents, zero pollution, circular economy, regulation

Rachel Carson’s *Silent Spring*,^1^ published just more than 60 years ago, outlined how the indiscriminate use of dichlorodiphenyltrichloroethane (DDT), a potent, environmentally persistent insecticide, was damaging the world’s ecosystems, animals, and food supply. There were many other chemicals more persistent than DDT accumulating in the environment when Carson was writing, including per- and polyfluoroalkyl substances (PFAS). While man-made, PFAS were not intended to cause harm, contrary to pesticides such as DDT. Today, ambient PFAS levels are contaminating rain, soil, and drinking water resources worldwide to such an extent that they have caused substantial, irreversible health and environmental damage.^2^ Like DDT, PFAS had long been in use by the time Rachel Carson was writing *Silent Spring* (see Figure 1). However, their environmental presence went unnoticed by Carson and other contemporary environmental researchers. PFAS were entering the environment under the radar, except to those who were manufacturing and emitting them.^3^

**Figure 1:**
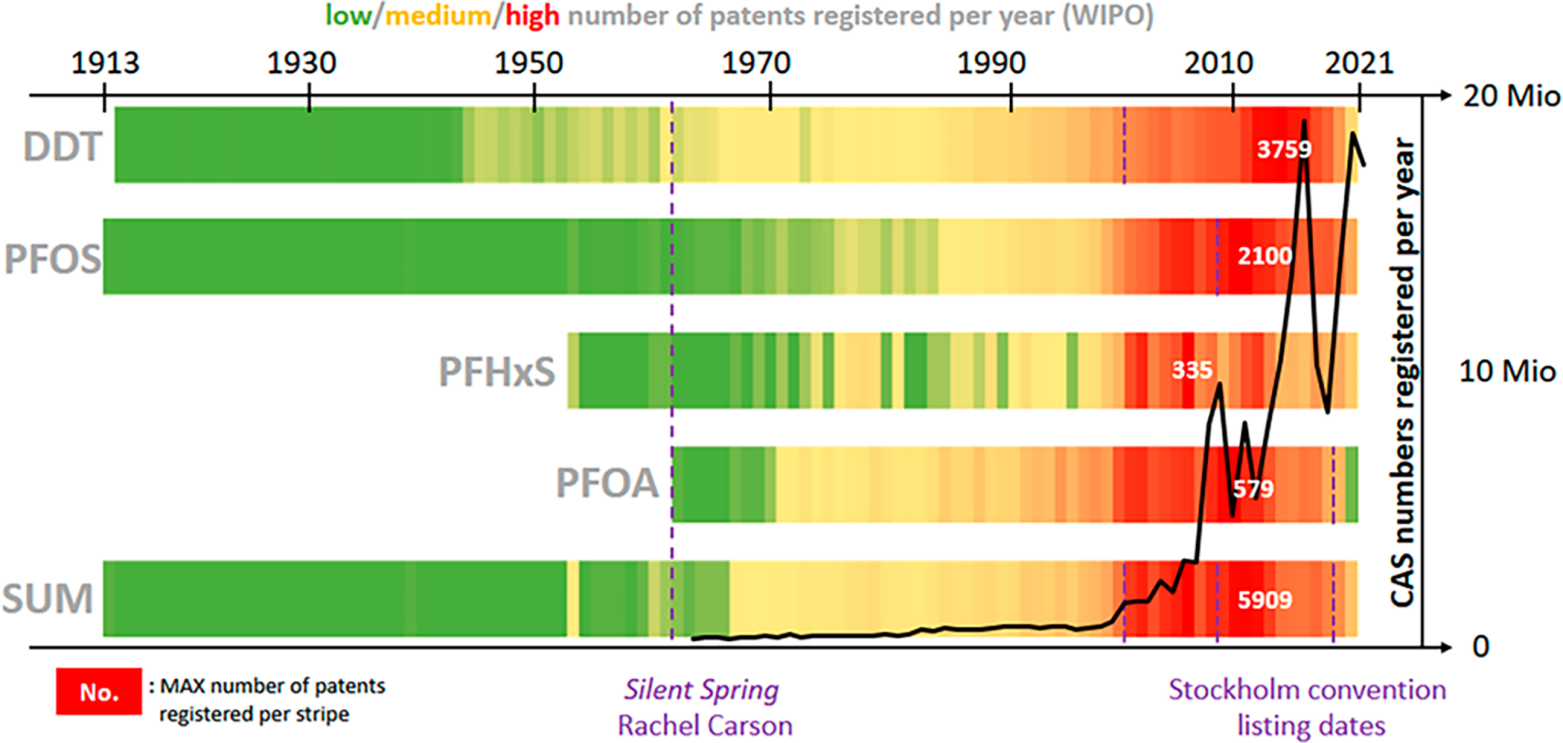
Chemical stripes for DDT and various PFAS. The colored stripes show the distribution of patents registered per year for DDT, PFOS, PFHxS, PFOA, and the sum of all four chemicals. Superimposed in black is the number of chemicals registered in the Chemical Abstracts Service (CAS) Registry each year. Purple dashes denote key publications and regulatory dates. Sources: World Intellectual Property Organisation (WIPO) patent numbers extracted from PubChem;^6^ CAS registration data provided by CAS.

## Why Were PFAS Not Considered by Rachel Carson?

When Rachel Carson was writing *Silent Spring*, the field of environmental chemistry was in its infancy, particularly in terms of the ability to detect synthetic organic substances in the environment. Carson’s case against excessive DDT use was triggered mainly by visible toxicological and ecological observations. Analytical data that proved ubiquitous exposure and accumulation in the food chain were lacking. Ultimately, it was James Lovelock’s development of electron capture detection and its coupling with gas chromatography^4^ that enabled other scientists to confirm the omnipresence and bioaccumulation of DDT. Lovelock reflected that the use of his technology to demonstrate the “ubiquitous distribution of pesticides throughout the global environment did much to fuel the environmental revolution which followed. [This] lent veracity to the otherwise unprovable statements of that remarkable book by Rachel Carson”.^4^

A few years after *Silent Spring*’s publication, Søren Jensen identified polychlorinated biphenyls (PCBs) in white-tailed eagle samples for the first time, while analyzing for DDT with this new technique.^5^ PCBs were later confirmed to be as ubiquitous in the environment and food chain as DDT. This discovery was rapidly accompanied by the detection of several other persistent organic pollutants (POPs), ultimately leading to the first “dirty dozen” POPs appearing in the United Nations Stockholm Convention, which was adopted in 2001, almost 40 years after Rachel Carson’s book was first published. By 2009, the most well-known substance of the PFAS family was added to the Stockholm Convention [perfluorooctanesulfonic acid (PFOS)]. Other PFAS have since followed, including perfluorooctanoic acid (PFOA) in 2019 and perfluorohexanesulfonic acid (PFHxS) in 2022 (Figure 1).

## What if Rachel Carson Had Mentioned PFAS in *Silent Spring*?

If Rachel Carson had known about PFAS and included them in *Silent Spring*, it is probable that the rapid global policy and industry action to manage DDT and PCBs would also have been applied to PFAS. One or more PFAS may even have been added to the original “dirty dozen” in 2001. Without the regulation or stewardship activities instigated by *Silent Spring*, there is little doubt that emissions of DDT, PFAS, and many other persistent pollutant groups would have been worse. This is evident in Figure 1, as the colored stripes present the relative number of filed patents for DDT and selected PFAS over time. The numbers of patents increased at an exponential rate, with patents for DDT and PFOS continuing to increase irrespective of regulatory efforts such as the Stockholm Convention listing dates shown in Figure 1. One exception to this is the most recent decrease in the number of patents for PFOA, which may be a sign of industry responding to its inclusion in the Stockholm Convention.

## Using Precaution to Prevent Future Silent Springs

A shortcoming of the implementation of a chemical regulation like the Stockholm Convention is that it is reactionary and not precautionary. Substances are added to the Stockholm Convention only after exposure and ecological harm has been demonstrated through environmental and laboratory observations, often long after the first awareness of red flags. But what of the other unknown environmentally persistent substances that are out there, or that may be in future?

Rachel Carson wrote in *Silent Spring*, “the new chemicals come from our laboratories in an endless stream; almost five hundred annually find their way into actual use in the United States alone. The figure is staggering and its implications are not easily grasped—500 new chemicals to which the bodies of men and animals are required somehow to adapt each year, chemicals totally outside the limits of biologic experience.”^1^ Shortly after *Silent Spring* was written, the number of chemicals present in the Chemical Abstract Services (CAS) Registry was 211 934 (in 1965). In March 2023, the total has reached 204 million chemicals, 3 orders of magnitude higher. In the past several years, the number of new CAS registrations has increased to 10–20 million per year (black line in Figure 1), 5 orders of magnitude higher than the rate of 500 chemicals per year quoted by Rachel Carson. Among the new CAS registrations are likely multiple extremely persistent substances, plausibly ranging from the hundreds to hundreds of thousands. Many of those being registered now could turn out to be the next DDT or PFAS.

The premise by the Renaissance physician Paracelsus, “the dose makes the poison”, is an irresponsible axiom for managing persistent substances that accumulate in the environment. In 1962, Carson did not know that PFAS could be a poison, and in 2023, scientists are still researching the dose.^2,7^ Because the number of chemicals registered per year (Figure 1) is now in the millions, it is clearly not possible or desirable to perform a detailed risk assessment for all substances. It is likely that there are already extremely persistent substances in the environment that are causing harm and for which we have little knowledge or data. So how can we stop this pattern of reoccurring silent springs?

## Improving the Management of Persistent Substances

A precautionary approach is the only way forward when it comes to managing new and existing, extremely persistent substances with a clear exposure pathway to humans and the environment. This precautionary approach must be applied to a future of chemical innovation that is centered around concepts such as the “circular economy” and “safe and sustainable by design” (SSbD), which consider the diverse impacts of chemicals over their entire life cycle.^8^ To enable this, research and innovation must shift toward making substances with lifetimes designed for their intended use within the circular economy. Such substances should degrade naturally or be triggered to do so at the end of their useful life cycle. As an illustrative example, some oxo-polymers are completely mineralizable in agricultural soils but can be persistent upon reaching marine environments.^9^ Such oxo-polymers are potentially safer replacements to extremely persistent pesticides and plastics on soils for which they were designed; however, containment to prevent marine emissions of these oxo-polymers at their end of life would become a management priority. Similarly, persistent substances found in reusable products would need to be managed such that they are either retained in the circular economy without emissions or designed for technical or natural degradation at the end of life.

To improve such management of persistent substances, there are three fronts that require further attention: improving experimental testing, developing *in silico* methods, and strengthening regulatory options. To improve experimental testing, simplified protocols that can be applied to several substances simultaneously would be highly valuable. This could include the development and implementation of “benchmarking” approaches, in which substances with unknown half-lives are placed in the same simulation system (or mesocosm) as those with well-known half-lives, and the degradation rates of the unknown substances are benchmarked to the known substances over time.^10,11^

Developing *in silico* methods will be necessary to strengthen and bridge the experimental and regulatory approaches to persistence. Considering the large number of chemicals on the global chemical market,^12^*in silico* methods are the only feasible way to assess all of them, though they remain highly inaccurate due to large data gaps.^13^ Nevertheless, high-quality *in silico* approaches, supported by additional experimental data, remain an aspiration as they require substantially less time and resources than experimental testing. In addition, *in silico* approaches could be used in the chemical design and synthesis phase to identify new and novel replacements for persistent substances for testing or development. Increasing the availability and digitization of high-quality experimental half-life and transformation data, coupled with advances in cheminformatics and machine learning tools, will increase the accuracy of *in silico* assessment of environmental persistence based on molecular structure.^14^

Finally, to improve regulatory options over the whole life cycle of chemicals, regulators could require and if necessary act upon information related to degradation conditions of substances, targeting chemical uses with pathways to the environment. Inspiration to improve such regulatory options can be found in a recent examination of approaches to persistence assessments^11^ and the European Union’s Chemicals Strategy for Sustainability (CSS).^15^ The CSS includes several initiatives to develop more precautionary approaches for extremely persistent substances, including a broad group restriction of PFAS, and the introduction of new hazard categories, including persistence [i.e., persistent, bioaccumulative, and toxic (PBT); very persistent and very bioaccumulative (vPvB); persistent, mobile, and toxic (PMT); and very persistent and very mobile (vPvM)].^15^

Expansion of work in these areas is required on a global scale to truly avoid the next silent spring, alongside the evolution and widespread adoption of approaches like SSbD and the circular economy. Improving our scientific understanding of environmental persistence, along with developing *in silico* methods, will encourage better, greener innovation and regulation that will result in the accumulation of fewer persistent substances in the environment. Learning from our past and present to improve a precautionary approach to persistent substances will ultimately allow humankind to foresee and forestall a future consigned to transgressing planetary boundaries and recurring silent springs.^1^
